# Aroma Properties of Cocoa Fruit Pulp from Different Origins

**DOI:** 10.3390/molecules26247618

**Published:** 2021-12-15

**Authors:** Thomas Bickel Haase, Ute Schweiggert-Weisz, Eva Ortner, Holger Zorn, Susanne Naumann

**Affiliations:** 1 Fraunhofer Institute for Process Engineering and Packaging IVV, Giggenhauser Straße 35, 85354 Freising, Germany; ute.weisz@ivv.fraunhofer.de (U.S.-W.); eva.ortner@ivv.fraunhofer.de (E.O.); 2 Institute of Food Chemistry and Food Biotechnology, Justus Liebig University Giessen, Heinrich-Buff-Ring 17, 35392 Giessen, Germany; holger.zorn@uni-giessen.de; 3 Institute for Nutritional and Food Sciences, University of Bonn, 53012 Bonn, Germany; 4 Fraunhofer Institute for Molecular Biology and Applied Ecology IME, 35392 Giessen, Germany

**Keywords:** *Theobroma cacao* L., by-product, aroma, aroma extract dilution analyses, gas chromatography-olfactometry, mass spectrometry

## Abstract

Cocoa pulp occurs as a by-product of cocoa bean production and can be repurposed to different food applications, such as jams, fruit preparations and beverages, improving the sustainability of cocoa production, as well as the livelihoods of cocoa farmers. In this work, aroma-active compounds of fresh cocoa fruit pulps from different origins were investigated by applying aroma extract dilution analyses in combination with gas chromatography-mass spectrometry/olfactometry for identification. In total, 65 aroma-active compounds were determined in four different pulps originating from Indonesia, Vietnam, Cameroon, and Nicaragua. Vietnamese pulp showed the highest number of aroma-active regions, while Cameroonian pulp accounted for the lowest. Moreover, Cameroonian cocoa pulp showed the lowest FD factors. Overall, the odorants with the highest FD factors were *trans*-4,5-epoxy-(*E*)-decenal, 2- and 3-methylbutanoic acid, 3-(methylthio)propanal, 2-isobutyl-3-methoxypyrazine, (*E*,*E*)-2,4-nonadienal, (*E*,*E*)-2,4-decadienal, 4-vinyl-2-methoxyphenol, *δ*-decalactone, 3-hydroxy-4,5-dimethylfuran-2(5*H*)-one, dodecanoic acid, and linalool. This study provides insights into the aroma composition of fresh cocoa pulp from different origins for future food applications.

## 1. Introduction

The International Cocoa Organization [1] forecasted for 2020/2021 a production of over 4.8 million tons of cocoa beans mainly destined for the chocolate industry. Taking into account the by-products of the cocoa bean processing chain, which represent together about 70–80% of the dry weight of the fruit and comprise the cocoa pod husk, the bean shells, and the pulp [2]; approximately 19 million tons of residual biomass will be produced. As the disposal of these by-products causes several social and environmental concerns, some studies have focused on the use of cocoa by-products in the formulation of new and versatile ingredients for the food, pharmaceutical, and cosmetic industries. By adding value to these side fractions, cocoa farmers may profit from new sources of income and better livelihoods, allowing also a more sustainable cocoa production [3,4]. Due to its pleasant flavor, cocoa fruit pulp has recently gained increasing attention of the food sector.

Cocoa pulp is a moist white fibrous layer that covers the fresh cocoa beans. With a low pH, usually ranging between 3.3 and 3.9, fresh cocoa pulp contains 83–86% water, 11–13% sugars (D-glucose, D-fructose, and sucrose), 0.5–1.2% pectin, 0.2–3% hemicelluloses, 0.7–0.9% cellulose, 0.1–0.3% lignin, and 0.3–1.3% citric acid [5,6,7]. The cocoa pulp is hydrolyzed during the fermentation of cocoa beans and flows out of the fermentation boxes as the so-called cocoa sweatings and is, thereby, lost [8]. Previous reports suggest that a fraction of the pulp can be separated from the fresh cocoa beans without having a negative effect on the later fermentation of the beans [9,10]. 

Accordingly, alternative applications for the use of the aromatic pulp are gaining popularity. Possible applications for the cocoa pulp, such as the production of cocoa jelly, alcohol, vinegar, nata, and processed juices, were described by Figueira et al. [11]. Moreover, Puerari et al. [12] took advantage of the high sugar content of cocoa pulp and used kefir grains for the development of an alcohol-containing drink, while dos Santos Filho et al. [13] inoculated cocoa juice with *Lactobacillus casei* for the production of a probiotic beverage. Further approaches focused on the use of cocoa pulp for wine production [14] and as an adjunct for the production of beer [15]. Additionally, the chocolate industry has used cocoa pulp as a replacement for added sugar and released chocolate-like products to the European and Asian market [16,17,18]. This broad range of pulp-based products highlights the high application potential of cocoa pulp in foods, which can be mainly attributed to its pleasant tropical flavor. 

To date, aroma research has primarily focused on the role of cocoa pulp for the later aroma quality of fermented cocoa beans. Pino et al. [19] studied the volatile composition of cocoa pulp from Colombia. The authors identified sixty-six different volatile organic compounds (VOCs) in the pulp, wherein the substances 2-heptyl acetate, 2-pentyl acetate, and linalool were present in the highest concentrations. Kadow et al. [20] quantified the VOCs of pulp and seeds of two fine flavor cocoas (SCA6, EET62) and a bulk cocoa (CCN51). Monoterpenes, present in SCA6, as well as methylketones, secondary alcohols, and their respective esters, present in EET62, were shown to be potential fine aroma components of cocoa beans originating in the pulp. Both studies investigated the cocoa pulp by means of headspace solid-phase microextraction gas chromatography mass spectrometry (HS-SPME CG-MS), yet did not investigate the aroma activity of the determined VOCs using olfactometric methods. Furthermore, to gain insights on how the cocoa pulp odorants affect the aroma quality of the beans during fermentation, Chetschik et al. [21] studied odor-active constituents in three cocoa pulp varieties by means of aroma extraction dilution analysis (AEDA) and gas chromatography-mass spectrometry/olfactometry (GC-MS/O). The experiments were performed with two cocoa varieties from Costa Rica (UF654 and CCN51) and one from Colombia (FSV41). In terms of flavor dilution (FD) factors, the CCN51 cocoa pulp presented lower aroma intensities than the varieties FSV41 and UF564, which showed more intense floral and fruity notes. By means of headspace solid-phase microextraction and GC-MS, Hegmann et al. [22] studied the influence of season and ripening stage on the cocoa pulp aroma of five cocoa varieties from Costa Rica. The total aroma diversity and intensity were shown to increase during ripening and aroma profiles were found to be more diverse when fruits ripened during the dry season, whereas the aroma intensity of the pulp was higher in the wet season. 

Several authors showed the influence of background (e.g., cocoa genotype, growing and harvesting conditions, and origin) as well as processing on the flavor of cocoa beans (e.g., fermentation, drying, and roasting); and how these factors determine the later quality of the cocoa beans [23,24,25,26,27,28]. In particular, the origin plays a decisive role on their final quality, as different countries cultivate botanically different subspecies of the cocoa tree. The cocoa cultivars can be divided into Criollo, Forastero, Trinitario, and Nacional [29,30]. Cocoa pulp is still an emerging market and little is known on how the origin of cocoa fruits determine the pulp’s flavor. In order to obtain cocoa pulps with constant and high qualities for the food sector, it is vital to study how the sourcing of cocoa pulp may affect its aroma composition and, thus, its suitability as a food ingredient. Investigations on the aroma-active compounds present in cocoa pulps from various origins and genetic backgrounds are a pre-requisite to identify possible application fields for the different cocoa pulps. Understanding the importance of certain aroma-active volatile organic compounds on the flavor of the cocoa pulps and elucidating the differences between the raw materials might help in the development of tailored food products for diverse markets and consumer groups. Therefore, the aim of this study was to investigate the differences in the main aroma-active compounds of four fresh cocoa pulps from different origins including comparative aroma extract dilution analysis (cAEDA) and GC-MS/O. In addition, the cocoa pulps were analyzed by HS-SPME CG-MS and stir-bar sorptive extraction gas chromatography-olfactometry/mass spectrometry (SBSE-GC-MS/O). Fresh cocoa pulps from South East Asia (Indonesia and Vietnam), Central America (Nicaragua), and Africa (Cameroon) were investigated in this study.

## 2. Results and Discussion

The aroma-active volatile compounds responsible for the aroma impression of cocoa pulps originating from Indonesia, Vietnam, Cameroon, and Nicaragua were isolated by extraction with dichloromethane (DCM) and separated from non-volatiles lipids using the solvent-assisted flavor evaporation (SAFE)-distillation technique (c.f. 3.2). The distillates obtained exhibited the typical characteristic overall aroma of each kind of pulp, proving the successful extraction of all key aroma compounds. The distillates were subjected to cAEDA by means of GC-O analyses. The cAEDA revealed a total of 65 aroma-active regions with an FD factor range of 2 to 1024 (Table 1). Of the detected aroma-active regions, five could not be conclusively identified. Substances identified by SBSE- and HS-SPME-GC-MS/O in the fresh pulps were also found in cocoa pulp distillates by GC-MS/O or did not exhibit aroma-active regions with FD > 2 in the cAEDA. Therefore, the results obtained with SBSE- and HS-SPME-GC-MS/O measurements are provided as Supplementary Materials. The identified aroma-active volatiles belonged to various chemical groups. Aldehydes were the most predominant group, followed by carboxylic acids, lactones, phenols, and ketones. Additionally, terpenes, alcohols, esters, pyrazines, furans, sulfides, and thiazolines were found in the sample distillates. Overall, 36 odorants could be perceived in all cocoa pulps (compounds **1**–**4**, **6**, **9**–**21**, **23**, **24**, **26**, **27**, **29**, **30**, **32**, **33**, **35**, **37**, **42**, **44**, **47**, **49**, **51**, **58**, **64**, and **65**). Out of the 65 identified aroma-active regions, 24 odorants (No. 1, 2, 5, 10, 11, 17, 18, 19, 21, 23, 28, 29, 30, 31, 32, 35, 37, 42, 44, 45, 47, 51, 54, and 65) were previously reported in cocoa pulp by means of AEDA [21]. In total, the authors identified 37 aroma-active regions with FD factors below FD 128. Overall, higher FD factors (up to FD 1024) were determined in this work. This may result from the fact that distillates were concentrated to a final volume of 100 µL, compared to 300 µL in the previous study. Moreover, compared to Chetschik et al. [21], more odorants were identified in this work, which may also be explained by differences in the innate aroma compositions of the investigated cocoa pulp varieties. 

In the investigated cocoa pulps, Vietnamese pulp displayed the highest number of aroma-active regions with a total of 57 odorants. It was followed by Nicaraguan cocoa pulp with 47, and Indonesian pulp with 46 aroma-active regions. Cocoa pulp originating from Cameroon accounted for a total of 43 aroma-active volatiles. Among others, all investigated cocoa pulps exhibited *fatty* (e.g., No. 19, 20, 26, 30, 32); *fatty*, *cheesy* (e.g., No. 24, 27, 29); *green*, *grassy* (e.g., No. 4, 6, 10, 13, 16); *flowery* (e.g., No. 12, 23, 37, 49, 58); *fruity* (e.g., 2, 33, 44); and *smoky* (e.g., No. 35, 51, 64) smelling compounds. The highest FD factors of 1024 and 512 were determined for 3-(methylthio)propanal (No. 18, *cooked potato-like*, RI 1455), 2-isobutyl-3-methoxypyrazine (No. 21, *bell pepper-like*, *earthy* RI 1510), linalool (No. 23; *flowery*, RI 1539), 2- & 3-methylbutanoic acid (No. 29 a/b, *rancid*, *cheesy*, RI 1662), (*E*,*E*)-2,4-nonadienal (No. 30, *fatty*, *deep fried*, RI 1696), (*E*,*E*)-2,4-decadienal (No. 32, *fatty*, RI 1800), *trans*-4,5-epoxy-(*E*)-2-decenal (No. 42, *metallic*, RI 1994), 4-vinyl-2-methoxyphenol (No. 51, *smoky*, *clove-like*, RI 2128), *δ*-decalactone (No. 53, *coconut-like*, RI 2188), 3-hydroxy-4,5-dimethylfuran-2(5H)-one (No. 54, *maggi-like*, *celery-like*, RI 2194), and dodecanoic acid (No. 63, *fatty*, *wax-like*, RI 2494). The aldehyde *trans*-4,5-epoxy-(*E*)-2-decenal was determined in all pulp samples with the highest FD factor (FD 1024). Correspondingly, Chetschik et al. [21] reported *trans*-4,5-epoxy-(*E*)-2-decenal with the highest FD factor in cocoa pulp from the varieties CCN51, FSV41, and UF654, which suggests an ubiquitous occurrence of this odorant in cocoa pulp. Due to the low odor threshold of *trans*-4,5-epoxy-(*E*)-2-decenal (6 × 10^−7^ µg/kg), very low concentrations can be determined by means of olfactometric methods [31]. Although cocoa pulp contains low amounts of fat (<0.8 g 100 g^−1^ fresh pulp) [6] and its fatty acid composition has not been yet elucidated, the presence of unsaturated fatty acids in cocoa pulp is probable, as these compounds have been reported in unroasted cocoa beans [32]. This may also explain the presence of the *fatty* and *wax-like* smelling odorant dodecanoic acid (No. 63, RI 2496) in cocoa pulp original from Nicaragua and Vietnam, as this fatty acid has been reported in cocoa butter [32]. Furthermore, as typical fatty acid degradation products, *peach-like* and *coconut-like* smelling lactones were perceived in all sample distillates (e.g., No. 44, 48, 52, 53). Lactones are formed from unsaturated fatty acids through several pathways, and often impart tropical aromas, whereas *δ*-lactones are less odor-active than *γ*-lactones [33,34]. While the *coconut-like* compound *γ*-nonalactone (No. 44, RI 2014) exhibited the highest intensity in pulp from Cameroon (FD 256), the related compound *δ*-nonalactone (No.48, *fruity*, *coconut-like*, RI 2084), which was identified in pulps of the other origins, was not detected. In addition, the odorants geraniol (No. 34, *flowery*, *earthy*; RI 1841), 3-hydroxy-4,5-dimethylfuran-2(5H)-one (No. 54, *maggi-like*, *celery-like*, RI 2194) and 3-propylphenol (No. 55, *medical*, RI 2247) were present in all but the Cameroonian cocoa pulp. The *butter-like* odorants 2,3-butandione (No. 1, RI 1008) and 2,3-pentandione (No. 3, RI 1056) were also determined in all samples (FD < 64), yet with higher intensities in Cameroonian pulp (FD 256). Similarly, the phenolic compound 4-methylphenol (No. 47, *fecal*, RI 2073) displayed the highest FD factor in pulp from Cameroon (FD 256). The *fruity* and *flowery* odorant 2-heptanone (No. 8, RI 1207) was shared only by samples from Indonesia and Cameroon with FD 16 and FD 32, respectively. This odorant has been described as more prevalent in cocoa pulp from ripe pods harvested during the rainy season [22]. Cameroonian cocoa pulp did not exhibit any exclusive odorants. It is of note that West Africa is responsible for about 70% of the total world production of cocoa and the largest producers are Côte d’Ivoire and Ghana, followed by Nigeria and Cameroon [35]. In West Africa, several subvarieties of the Forastero cocoa type are produced. While the Amelonado cocoa, a subvariety of the Forastero type, is extensively cultivated in most West African countries, the hybrid Trinitario, a mix of Criollo with Forastero, is the predominant variety in Cameroon [36,37]. Moreover, even if the terms fine or flavor cocoas are often used to describe the cocoa qualities, there is no agreed definition of the terms, except that these cocoas are sold at a premium price for their flavor. Fine or flavor cocoas can exhibit attributes that are often described as *fruity*, *raisin*, *brown fruit*, *floral*, *spicy*, *aromatic*, *nutty*, *molasses-like*, and *caramel* [38]. Generally, these cocoas come from Criollo, Trinitario, or Nacional-type trees. Nonetheless, not all cocoas of these varieties can be considered as fine or flavor [36]. For instance, Nacional trees in Ecuador, considered to be Forastero type trees, produce fine or flavor cocoa, yet cocoa beans from Cameroon, produced by Trinitario type trees have, hitherto, been classified as bulk cocoa beans [39]. It is therefore reasonable to assume that the Cameroonian cocoa pulp analyzed in this work, would be considered as bulk cocoa.

With regard to aroma diversity, the intensity of the *flowery* smelling linalool showed a lower FD in Indonesian pulp (FD 32) compared to the other samples (FD 512). This odorant is thought to be a *fine flavor* aroma compound in cocoa beans, where it is present in higher concentrations compared to bulk cocoa [40]. It has been also proposed that certain aroma compounds present in the cocoa pulp, including the odorant linalool, may originate in the pulp and migrate to the beans during cocoa bean fermentation [41]. In addition, odorants of the group of phenols were identified in this work. The compound 4-vinyl-2-methoxyphenol (No. 51, *smoky*, *clove-like*, RI 2128) was found in all cocoa pulp samples with similar FD factors. The odorant 2,3-dimethylphenol (No. 50, *phenolic*, RI 2109) was exclusively found in Indonesian pulp with factor FD 256. On the other hand, the unknown substance No. 57 (RI 2341), which exhibited a *smoky* and *phenolic* odor quality, was only found in pulp from Vietnam. Phenols can be released by the degradation of polyphenols and lignin by enzymes and microbes [42]. Phenols and their derivatives can be potent odor-active compounds. Methylphenols usually impart *phenolic* and *smoky* odor qualities, while methoxyphenols are often described with a broad range of aroma qualities such as *smoky*, *vanilla*, *leather*, *meat*-, and *ham-like*. Terpene-derived phenols can be *herbal* and *spice-like* odorants [34]. Further odorants present only in Indonesian cocoa pulp were the lactone coumarin (No. 61, *cinnamon-like*, RI 2435, FD 256) and the heterocyclic compound indole (No. 62, *fecal*, RI 2485, FD 16). Coumarin can be found naturally in many food products such as fruits, oils, nuts, and spices [42], and was identified by Balladares et al. [43] in cocoa pulp sweatings from Ecuador. Additionally, Mashuni et al. [44] found coumarin derivatives in the polyphenol fraction obtained from cocoa pod husks by means of a microwave-assisted extraction method. The compound indole is a nitrogen-containing odorant that imparts a floral taint with a fecal note to food products [24] and is often produced by bacteria [45]. Indonesia is mainly a bulk cocoa producer and only 1% of its total cocoa exports is classified as fine and flavor cocoa [46]. Therefore, it is likely that the analyzed SUL2 variety, categorized as a Trinitario cocoa [47,48], would be also considered to have a bulk cocoa quality. Altogether, the low FD factor of linalool in Indonesian cocoa pulp and the *fatty*, *cheesy*, and *phenolic* notes suggest that the pulp from this origin, specifically of the variety SUL2, is less suitable for products in which predominant attributes such as *fresh*, *fruity*, and *floral* are desired (e.g., juices, jams, fruit snacks). 

Cocoa pulp from Vietnam also showed unique aroma compounds. The substances (*E*,*Z*)-2,6-nonadienal (No. 25, *cucumber-like*, *fatty*, RI 1574, FD 64), ethyl (*E*,*E*)-2,4-decadienoate (No. 36, *metallic*, *pear-like*, RI 1890, FD 128), *γ*-octalactone (No. 38, *fruity*, *coconut-like*; RI 1908, FD 4), 4-methylhexanoic acid (No. 43, *sweaty*, *fishy*, RI 2011, FD 8), 4-hydroxy-2,5-dimethyl-3(2*H*)-furanone (No. 45, *caramel-like*, RI 2026, FD 64), *γ*-decalacton (No. 52, *fruity*, *peach-like*, RI 2133, FD 16), undecanoic acid (No. 56, *soapy*, *coriander-like*, RI 2323, FD 64), substance No. 57 (unknown, *smoky*, *phenolic*, RI 2341, FD 1024), and *δ*-dodecalactone (No. 60, *peach-like*, RI 2393, FD 16) could not be determined in Indonesian, Cameroonian, and Nicaraguan cocoa pulp. The odorant 4-hydroxy-2,5-dimethyl-3(2*H*)-furanone was first identified as a Maillard reaction product [49]. In addition, it has been determined in strawberries [50], pineapples [51], tomatoes [52], grapes [53], and raspberries [54]. Chetschik et al. [21] identified 4-hydroxy-2,5-dimethyl-3(2*H*)-furanone (furaneol) in cocoa pulp from Colombia and Costa Rica. Furthermore, Vietnamese pulp displayed the most perceivable odorants from the group of lactones (*n* = 7) compared to the three counterparts (*n* < 5). However, lactones were determined with low FD factors, accentuating the predominance of odorants with *green*, *fatty*, and *smoky* notes. Moreover, compared to other varieties, the odorant 2-isobutyl-3-methoxypyrazine (No. 21, *bell pepper-like*, *earthy*, RI 1510) was more intense (FD 512) in the South East Asian pulp. This pyrazine is characteristic for bell peppers [55] and certain types of grapes [56]. Vietnamese pulp also shared odorants with other single samples. The odorant 3-methylbutanol (No. 7, RI 1200), which exhibits *malty* and *roasty* odor qualities, was only perceived in Vietnamese and Nicaraguan pulp.

Odorants (*E*)-2-nonenal (No. 22, *fatty*, *cardboard-like*, RI 1524), compound No. 39 (*metallic*, RI 1920), and octanoic acid (No. 46, *green*, *soapy*, RI 2043) were found only in pulp from Vietnam and Cameroon. Although cocoa pulps from Vietnam and Indonesia came both from South East Asia and were expected to show strong resemblances in their aroma compositions, they did not share odorants exclusively. ICCO recognized that 40% of the total cocoa exports of Vietnam can be classified as fine and flavor cocoa [46]. Along with this, the predominance of *green*, *fatty*, and *smoky* notes suggests that Vietnamese cocoa pulp investigated in this work originated from a bulk cocoa variety.

The cocoa variety TSH 565, short for Trinidad Selected Hybrid 565, was obtained from the crossing of varieties ICS1 (Imperial College Selection 1) and SCA6 (Scavina 6). While the variety ICS1 has been reported to be original to Trinidad as well as to produce superior beans [57], Scavina cocoa varieties originated from the Ucayali River basin [58]. Scavina cocoa is known for its floral aroma notes in the fruit pulp and raw cocoa [20]. The resulting hybrid TSH 565 can be associated to fine flavor cocoa due to its high concentrations of terpenes such as β-myrcene and β-cis-ocimene [59]. Compared to other origins investigated in this work, cocoa pulp from the TSH 565 variety grown in Nicaragua exhibited higher FD factors in aroma compounds with the odor qualities *fruity* and *flowery*. For instance, the odorant 3-methylbutyl acetate (No. 5, *fruity*, RI 1118) was determined with FD 128 in Nicaraguan, FD 16 in both South East Asian samples, and FD < 2 in Cameroonian pulp. In addition, the odorant (*E*)-2-heptenal (No. 12, *green, flowery*, RI 1311) displayed an FD factor of 128 in Nicaraguan, 16 in Indonesian and Cameroonian, and 32 in Vietnamese cocoa pulp. The aldehyde nonanal (No. 15, *citrus-like, soapy*, RI 1376) showed FD factors of 256, 8, 32, and 32, respectively. The odorants (*E*)-2-heptenal and nonanal have been reported previously in cocoa pulp from Colombia [19], but could not be found in five cocoa pulp varieties from Costa Rica [22]. Furthermore, cocoa pulp from Nicaragua stood out for having the lower intensity of acetic acid (FD 16) compared to the Indonesian (FD 128), Vietnamese (FD 128), and Cameroonian (FD 256) counterparts. Odorant No. 53, *δ*-decalactone (*coconut-like*, RI 2188), was only perceived in Cameroonian and Nicaraguan cocoa pulp (FD 128 and FD 512, respectively). The odorants phenylacetaldehyde (No. 28, *flowery*, *honey-like*, RI 1634, FD 32) as well as 2-methoxy-4-methylphenol (No. 41, *clove-like, vanilla-like*, RI 1962; FD 128) were also exclusive for pulp from Nicaragua (FD 32). Together with the *honey-like* phenylacetic acid (No. 65, RI 2545, FD 256), the odorants may have contributed to the overall *honey-like* impression of the Nicaraguan extracts. The combination of *fruity, flowery, honey-like,* and *vanilla-like* odor qualities of Nicaraguan cocoa pulp make it interesting for the development of food products. The results may possibly relate to the fact that Nicaragua is solely a fine flavor cocoa exporter [46]. An industrial implementation as well as the existing infrastructure in the country would have to be revised, as Nicaragua is a small cocoa producer compared to countries such as Indonesia and Cameroon [60,61].

## 3. Materials and Methods

### 3.1. Separation and Storage of the Fresh Cocoa Pulp

Cocoa pulps from Indonesia (SUL2), Cameroon (unknown variety), Vietnam (unknown variety), and Nicaragua (TSH 565) were investigated. Cameroonian, Vietnamese, and Nicaraguan fresh cocoa fruits, harvested in 2019, were imported to Germany in a cool shipment directly after harvest. At Fraunhofer IVV, the fruits were washed, cut open and the cocoa pulp was separated mechanically immediately upon arrival. After de-pulping, the pulp was vacuum-sealed in odorless plastic bags (PA/PE 90/130 × 280 mm, Dagema eG, Willich, Germany) and immediately frozen at −50 °C. Indonesian cocoa pulp was supplied by the Indonesian Cocoa and Coffee Research Centre (ICCRI) in Jember, East Java. The cocoa pulp was separated mechanically on-site (Indonesia) directly after cocoa pod harvest (wet season 2019) and frozen immediately. The material was shipped in a frozen state to Germany, where it was stored at −50 °C in the same manner as its counterparts.

### 3.2. Isolation of Volatile Organic Compounds (VOC)

To isolate the volatile organic compounds, 50 g (±0.1) fresh cocoa pulp was extracted by stirring vigorously with 200 mL dichloromethane (DCM) for one hour at room temperature in a closed vessel. Dichloromethane (DCM) was purchased from Merck KGaA (Darmstadt, Germany) and distilled for purification prior to use. After decanting of 150 mL DCM, the volatiles were separated from the non-volatiles by means of the Solvent Assisted Flavor Evaporation (SAFE) technique [62]. The distillation was carried out under high vacuum, maintaining the round flask in a water bath at 50 °C and thermostating the SAFE apparatus to 55 °C. The obtained distillates were dried over anhydrous sodium sulfate (Merck KGaA, Darmstadt, Germany), filtered, and concentrated at 50 °C to ~3 mL by a Vigreux column (50 cm × 1 cm i.d.) and finally to a volume of ~100 μL by microdistillation [63]. To enable a comparison between the pulps, the same amounts were extracted, subjected to SAFE distillation, concentrated to the same final volume, and, finally, the same volume was used for GC-O.

### 3.3. Comparative Aroma Extract Dilution Analysis (cAEDA)

To enable a comparison between all pulps, the same volumes of the extracts were used for gas chromatography-olfactometry (GC-O). To avoid a potential overlooking of aroma-active areas, the original distillates were evaluated by four trained panelists of the Fraunhofer IVV sensory panel using GC-O. The panelists undergo trainings once a week on an in-house established odor-language with selected reference compounds, corresponding to about 150 different odorants. All panelists exhibited normal olfactory function and had no known illnesses at the time of examination. A blank was performed for each sample by applying the same work-up procedure (c.f 3.2). The flavor dilution (FD) factors of the odorants were determined by diluting the distillate stepwise (1 + 1, *v* + *v*) with dichloromethane up to factor 1024 and analyzing the dilutions with GC-O [64]. For each aroma-active area, the respective FD factor, correlating to the highest dilution in which the compound was perceived by the panelists at the odor detection port for the last time, was assigned.

### 3.4. Gas Chromatography-Olfactometry (GC-O)

GC-O was carried out by means of a Trace GC Ultra (Thermo Fisher Scientific GmbH, Dreieich, Germany) equipped with either a DB-FFAP or DB-5 capillary column (both 30 m × 0.32 mm, 0.25 μm film thickness) (J&W Scientific, Agilent Technologies GmbH, Waldbronn, Germany). Aliquots (2 µL) of the sample distillates were injected manually by the cold on-column technique at 40 °C, using helium as carrier gas at a constant flow mode (2.2 mL/min). The initial temperature of 40 °C was held for 2 min, raised at 6.0 °C/min to 235 °C (DB-FFAP) or 250 °C (DB-5), respectively, and held for 5 min. At the end of the column, the effluent was split 1:1 by volume through two deactivated fused silica capillaries of the same length (70 cm × 0.2 mm) leading to a flame ionization detector (FID) held at 250 °C and to an odor detection port (ODP) held at 235 °C. Linear retention indices (RI) were calculated using a homologous series of n-alkanes ranging from C_6_ to C_26_ for the DB-FFAP and C_6_ to C_18_ for the DB-5 column [65].

### 3.5. Gas Chromatography-Mass Spectrometry/Olfactometry (GC-MS/O)

For identification of the VOCs present in the distillates, GC-MS/O was performed using a Trace GC Ultra and a Trace dual stage quadrupole (DSQ) mass spectrometer (both Thermo Fisher Scientific GmbH) equipped with a DB-FFAP column (30 m × 0.32 mm, 0.25 μm film thickness, J&W Scientific, Waldbronn, Germany). The distillates (2 µL) were injected automatically by a multipurpose autosampler MPS 2 (Gerstel GmbH & Co. KG, Mülheim an der Ruhr, Germany) using the cold on-column technique. The initial temperature of 40 °C was held for 2 min, raised at 6.0 °C/min to 235 °C, and held for 5 min. The flow rate of the helium carrier gas was 2.2 mL/min. At the end of the capillary column, the effluent was split between an ODP and the MS using deactivated fused silica capillaries (50 cm × 0.2 mm). Mass spectra were recorded in positive electron ionization (EI) mode at 70 eV (*m*/*z* range 35–250).

### 3.6. Stir-Bar Sorptive Extraction Gas Chromatography-Olfactometry/Mass Spectrometry (SBSE-GC-MS/O)

The SBSE method was applied to confirm the presence of VOCs and to compensate for possible losses and changes in the aroma composition of the pulp during the isolation step (c.f. 3.2). Fresh cocoa pulp samples (2.0 g ± 0.01) were diluted individually 1:1 (*w*/*w*) in distilled water in headspace vials (volume 20 mL), sealed airtight, and the suspension was stirred at room temperature for 15 min with a preconditioned SBSE Twister^®^ (polydimethylsiloxane sorbent (PDMS), 20 mm length, 0.5 mm coating thickness; Gerstel GmbH & Co. KG, Mülheim an der Ruhr, Germany). Preconditioning was performed at 280° C for five hours. After extraction, the Twister^®^ was automatically transferred to the Thermal Desorption Unit at 40 °C (TDU, Gerstel GmbH & Co. KG, Mülheim an der Ruhr, Germany). Desorption started at a temperature of 40 °C (initial time: 0.1 min), then increased to 250 °C at a rate of 12.0 °C/s, before being held at 250 °C for 5 min. The desorbed volatiles were transferred to the cold-injection-system (CIS, Gerstel GmbH & Co. KG, Mülheim an der Ruhr, Germany), cooled at −70 °C using liquid nitrogen, and then transferred onto the GC-MS/O system (cf. Section 3.5) by thermal desorption. After an initial time of 2 min at 40 °C, the oven temperature was raised at 6 °C/min to 235 °C and held for 5 min. The column flow of the helium carrier gas was adjusted to 50 mL/min. Mass spectra were generated in full scan mode (*m*/*z* range 35–400, EI 70 eV) on the same GC-MS system as described in Section 3.5.

### 3.7. Headspace Solid-Phase Microextraction Gas Chromatography-Olfactometry/Mass Spectrometry (HS-SPME GC-O/MS)

Highly volatile aroma-active compounds were additionally analyzed using HS-SPME-GC-O/MS to rule out any losses during the concentration steps by the liquid extraction technique. Fresh cocoa pulp samples (1.0 g ± 0.01) were diluted individually 1:1 (*w*/*w*) in distilled water and air tightly sealed in a 20 mL headspace glass vial. By means of an orbital shaker (Gerstel GmbH & Co. KG, Mülheim an der Ruhr, Germany), each vial was agitated for 10 min at 50 °C and 250 rpm, changing the direction every 90 s. Prior to use, the SPME fiber (PDMS, 100 µm, Supelco, Bellefonte, PA, USA) was conditioned at 250 °C for 5 min. The SPME fiber was introduced automatically into the vial for volatile adsorption, exposed to the fresh cocoa pulp for 10 min at 50 °C during HS equilibration, and analyzed using the MPS autosampler and GC-O/MS system equipped with a DB-FFAP capillary column as described in Section 3.5. Thermal desorption was performed using a PTV (Gerstel GmbH & Co. KG, Mülheim an der Ruhr, Germany) for 180 s at 250 °C. After an initial time of 2 min at 40 °C, the GC-oven temperature was raised at 6 °C/min to 150 °C, then raised at 20 °C/min to 235 °C and held for 5 min. The column flow of the helium carrier gas was adjusted to 50 mL/min. Mass spectra were generated in full scan mode (*m*/*z* range 35–250, EI 70 eV).

## 4. Conclusions

Our study shows that cacao fruit pulp may present great qualitative differences in the composition of volatiles depending on their origin, which then may determine their suitability for diverse types of food products. By means of cAEDA, 65 aroma-active areas were detected in cocoa pulps from various origins, 60 of which were unequivocally identified by GC analysis on two columns of different polarity and comparison to authentic reference standards. Of the identified compounds, 36 were found in all four cocoa pulps. The highest odorant diversity was found in Vietnamese pulp. Cocoa pulp from Cameroon exhibited the lowest flavor intensities in terms of FD factors. In the higher dilutions, the Indonesian cocoa pulp extract was predominantly *fatty*, *cheesy*, and *phenolic*. The Vietnamese cocoa pulp extract displayed high FD factors with *fatty*, *green*, and *smoky* notes. Cameroonian cocoa pulp presented *butter-like*, *popcorn-like*, *flowery*, and *fruity* odor qualities in the higher extract dilutions, whereas the Nicaraguan cocoa pulp extract was primarily *fruity*, *flowery*, but also possessed *honey*, *clove*, and *vanilla-like* traits. Given that odor impressions result from complex interactions between many aroma-active substances, correlations between a single volatile and an olfactory perception are often not conclusive [66]. Synergistic, additively or suppressive effects between substances present in the sample may affect its overall aroma impression unpredictably [66,67]. In order to understand the final aroma as well as the aroma compound diversity in cocoa pulp better, a quantification of the VOCs would be needed. Furthermore, the influence of genetic background on the aroma quality of the cocoa pulp should be investigated, as the genetic backgrounds of samples analyzed in this work were unclear. Studies with bigger cocoa pulp cohorts could provide important insights on how cocoa genetics determine the aroma composition of cocoa pulps. Finally, investigations using one commercial cocoa genotype, e.g., CCN51, grown in different regions of the world could help us understand better how other factors, such as the harvesting season, age of the cocoa tree, and growing conditions, may affect the aroma quality of cocoa pulp and its later application as a food ingredient.

## Figures and Tables

**Table 1:** Aroma-active compounds identified in extracts of cocoa pulps grown in Indonesia, Vietnam, Cameroon, and Nicaragua.

			Retention Index on		FD Factor ^d^			
No. ^a^	Odorant ^b^	Odor Quality ^c^	DB-FFAP	DB-5	Indonesia	Vietnam	Cameroon	Nicaragua
1	2,3-butandione	butter-like	1008	731	64	16	256	16
2	methyl 2-methylbutanoate	fruity, banana-like	1017	776	4	32	4	4
3	2,3-pentanedione	butter	1056	706	64	16	256	16
4	hexanal	green	1089	802	4	128	32	32
5	3-methylbutyl acetate	fruity	1118	880	16	16	<2	128
6	*δ*-carene	green	1140	1014	2	32	8	64
7	3-methylbutanol	malty, roasty	1200	760	<2	32	<2	8
8	2-heptanone	fruity, flowery	1207	891	16	<2	32	<2
9	*(Z)*-4-heptenal ^f^	fishy	1245	894	32	128	8	32
10	octanal	citrus-like, green	1280	1002	256	128	64	64
11	1-octen-3-one	mushroom-like	1285	978	32	128	16	128
12	*(E)*-2-heptenal	green, flowery	1311	951	16	32	16	128
13	1-hexanol	green, grassy	1338	n.d. ^e^	2	128	128	32
14	2-acetyl-1-pyrroline ^f^	popcorn-like	1342	930	64	128	128	32
15	nonanal	citrus-like, soapy	1376	1106	8	32	32	256
16	*(E)*-2-octenal	fatty, grassy, green	1417	1055	128	128	64	128
17	acetic acid	vinegar-like	1430	n.d. ^e^	128	128	256	16
18	3-(methylthio)propanal	cooked potato-like	1455	903	64	128	64	512
19	(*E*,*E*)-2,4-heptadienal	fatty, roasty	1486	1020	4	32	128	32
20	(*Z*)-2-nonenal	green, fatty	1494	1140	256	256	128	128
21	2-isobutyl-3-methoxypyrazine	bell pepper -like, earthy	1510	1090	2	512	32	4
22	*(E)*-2-nonenal	fatty, cardboard-like	1524	1164	<2	128	128	<2
23	linalool	flowery	1539	1103	32	512	512	512
24	2-methylpropanoic acid	cheesy	1562	782	256	128	256	64
25	(*E*,*Z*)-2,6-nonadienal	cucumber-like, fatty	1574	1158	<2	64	<2	<2
26	*(E)*-2-decenal	green, fatty	1616	1252	16	128	64	128
27	butanoic acid	cheesy	1626	804	128	256	32	4
28	phenylacetaldehyde	flowery, honey-like	1634	1040	<2	<2	<2	32
29 a/b	2- and 3-methylbutanoic acid	rancid, cheesy	1662	860	1024	128	128	128
30	(*E*,*E*)-2,4-nonadienal	fatty, deep fried	1696	1216	512	256	64	512
31	2-acetyl-2-thiazoline ^f^	popcorn-like, roasty	1747	n.d. ^e^	16	256	32	<2
32	(*E*,*E*)-2,4-decadienal	fatty	1800	1325	64	128	512	256
33	*ß*-damascenone ^f^	fruity, grape-like	1808	1374	2	256	64	128
34	geraniol	flowery, earthy	1841	1428	64	64	<2	16
35	2-methoxyphenol	smoky, ham-like	1848	1096	16	64	128	128
36	ethyl (*E*,*E*)-2,4-decadienoate ^f^	metallic, pear-like	1890	n.d. ^e^	<2	128	<2	<2
37	2-phenylethanol	rose-like, flowery	1897	1110	32	128	128	32
38	*γ*-octalactone	fruity, coconut-like	1908	1154	<2	4	<2	<2
39	unknown	metallic	1920	n.d. ^e^	<2	128	128	<2
40	unknown	metallic	1947	1545	<2	16	<2	<2
41	2-methoxy-4-methylphenol	clove-like, vanilla-like	1962	1198	<2	<2	<2	128
42	*trans*-4,5-epoxy-(*E*)-2-decenal	metallic	1994	1379	1024	1024	512	1024
43	4-methylhexanoic acid ^f^	sweaty, fishy	2011	n.d. ^e^	<2	8	<2	<2
44	*γ*-nonalactone	fruity, coconut-like	2014	1364	16	64	256	8
45	4-hydroxy-2,5-dimethyl-3(2*H*)-furanone	caramel-like	2026	1080	<2	64	<2	<2
46	octanoic acid	green, soapy	2043	1180	<2	32	4	<2
47	4-methylphenol	fecal	2073	1085	64	32	256	8
48	*δ*-nonalactone	fruity, coconut-like	2084	1380	32	64	<2	128
49	unknown	flowery, earthy	2103	n.d. ^e^	4	64	32	2
50	2,3-dimethylphenol	phenolic	2109	1200	256	<2	<2	<2
51	4-vinyl-2-methoxyphenol	smoky, clove-like	2128	1326	256	128	512	256
52	*γ*-decalactone	fruity, peach-like	2133	1474	<2	16	<2	<2
53	*δ*-decalactone	coconut-like	2188	1507	<2	<2	128	512
54	3-hydroxy-4,5-dimethylfuran-2(5*H*)-one	maggi-like, celery-like	2194	1106	16	512	<2	128
55	3-propylphenol	medical	2247	1285	64	128	<2	256
56	undecanoic acid	soapy, coriander-like	2323	1475	<2	64	<2	<2
57	unknown	smoky, phenolic	2341	1345	<2	1024	<2	<2
58	*γ*-dodecalactone	caramel-like, flowery	2371	1667	256	128	32	64
59	4-methoxyphenol	phenolic	2388	1071	<2	<2	<2	16
60	*δ*-dodecalactone	peach-like	2393	1700	<2	16	<2	<2
61	coumarin	cinnamon-like	2435	1440	256	<2	<2	<2
62	indole	fecal	2485	1320	16	<2	<2	<2
63	dodecanoic acid	fatty, wax--like	2496	1574	<2	1024	<2	256
64	unknown	smoky, flowery	2516	n.d. ^e^	64	256	128	16
65	phenylacetic acid	honey-like	2545	1256	16	128	64	256

^a^ Consecutive numbering of odorants according to their retention indices on capillary column DB-FFAP. ^b^ Odorant was identified by comparison of its odor quality and intensity and retention indices on capillaries DB-FFAP and DB-5 as well as mass spectra (EI mode) with data of reference compounds. ^c^ Odor quality perceived at the odor detection port by four trained panelists. ^d^ Flavor dilution factor determined on DB-FFAP. ^e^ n.d. not detected. ^f^ No unequivocal mass spectrum was obtained; identification is based on the remaining criteria given in footnote b.

## Data Availability

The data presented in this study are available on request from the corresponding author.

## Supplementary Materials

The following are available online: results obtained by HS-SPME-GC-MS/O and SBSE-GC-MS/O.

## Sample Availability

Samples of the compounds are not available from the authors.
