# Supplementary material for: Aroma Properties of Cocoa Fruit Pulp from Different Origins
Source: Molecules. 2021 Dec 15;26(24):7618. doi: 10.3390/molecules26247618 (PMC8704363; doi:10.3390/molecules26247618)
Supplement: Supplementary file 1 [file molecules-26-07618-s001.zip › molecules-1489637-supplementary.pdf]

Table S1: Aroma-active compounds identified in cocoa pulps grown in Indonesia, Vietnam, Cameroon and Nicaragua by means of SBSE-GC-MS

				**retention index on**
No. [a]	Odorant [b]	Molecular weight [g/mol]	Odor Quality [c]	DB-FFAP
1	methyl 2-methylbutanoate	116.16	fruity, banana-like	1017
2	hexanal	100.16	green	1089
3	(-)-beta-pinene [d]	136.23	green, eucalyptus-like	1101
4	3-methylbutyl acetate	130.18	fruity	1118
5	δ-carene	136.23	green	1140
6	R-(+)-limonene [d]	184.23	citrus-like	1189
7	3-methylbutanol	88.15	malty, roasty	1200
8	2-heptanone	114.19	fruity, flowery	1207
9	pentan-1-ol [d]	88.15	fruity	1241
10	octanal	128.21	citrus-like, green	1280
11	(*E*)-2-heptenal	112.17	green, flowery	1311
12	1-hexanol	102.17	green, grassy	1338
13	nonanal	142.24	citrus-like, soapy	1376
14	(*E*)-2-octenal	126.2	fatty, grassy, green	1417
15	acetic acid	60.05	vinegar-like	1430
16	(*E*,*E*)-2,4-heptadienal	110.15	fatty, roasty	1486
17	(Z)-2-nonenal	140.22	green, fatty	1494
18	benzaldehyde [d]	106.12	almond-like	1515
19	(*E*)-2-nonenal	140.22	fatty, cardboard-like	1524
20	linalool	154.25	flowery	1539
21	1-octanol [d]	130.23	soapy, green	1545
22	(*E*,*Z*)-2,6-nonadienal	138.21	cucumber-like, fatty	1574
23	(*E*)-2-decenal	154.25	green, fatty	1616
24 a/b	2- and 3-methylbutanoic acid	102.13	rancid, cheesy	1662
25	(*E*,*E*)-2,4-nonadienal	138.21	fatty, deep fried	1696
26	pentanoic acid [d]	102.13	fruity	1702
27	(*E*,*E*)-2,4-decadienal	152.23	fatty	1800
28	2-methoxyphenol	124.14	smoky, ham-like	1848
29	2-phenylethanol	122.16	rose-like, flowery	1897
30	4-hydroxy-2,5-dimethyl-3(2*H*)-furanone	128.13	caramel-like	2026
31	2,3-dimethylphenol	122.16	phenolic	2109
32	4-vinyl-2-methoxyphenol	150.17	smoky, clove-like	2128
33	3-hydroxy-4,5-dimethylfuran-2(5*H*)-one	128.13	maggi-like, celery-like	2194
34	undecanoic acid	186.29	soapy, coriander-like	2323
35	4-methoxyphenol	124.14	phenolic	2388
36	dodecanoic acid	200.32	fatty, wax-like	2496
37	phenylacetic acid	136.15	honey-like	2545

[a] Consecutive numbering of odorants according to their retention indices on capillary column DB-FFAP. [b] Odorant was identified by comparison of its odor quality and intensity, retention indices on capillary DB-FFAP as well as mass spectra (EI mode) with data of reference compounds. [c] Odor quality perceived at the odor detection port. [d] Odorant not determined in the cocoa pulp extracts with a factor of FD>2

Table S2: Aroma-active compounds identified in cocoa pulps grown in Indonesia, Vietnam, Cameroon and Nicaragua by means of HS-SPME-GC-MS

No. [a]	Odorant [b]	Molecular weight [g/mol]	Odor Quality [c]	**retention index on** DB-FFAP
1	2,3-pentanedione	100.12	butter	1056
2	hexanal	100.16	green	1089
3	(-)-beta-pinene [d]	136.23	green, eucalyptus-like	1101
4	3-methylbutyl acetate	130.18	fruity	1118
5	p-xylene [d]	106.16	green	1130
6	δ-carene	136.23	green	1140
7	myrcene [d]	136.23	earthy, geranium-like	1160
8	R-(+)-limonene [d]	184.23	citrus-like	1189
9	3-methylbutanol	88.15	malty, roasty	1200
10	2-heptanone	114.19	fruity, flowery	1207
11	pentan-1-ol [d]	88.15	fruity	1241
12	octanal	128.21	citrus-like, green	1280
13	*(E)*-2-heptenal	112.17	green, flowery	1311
14	1-hexanol	102.17	green, grassy	1338
15	nonanal	142.24	citrus-like, soapy	1376
16	*(E)*-2-octenal	126.2	fatty, grassy, green	1417
17	acetic acid	60.05	vinegar-like	1430
18	(*E*,*E*)-2,4-heptadienal	110.15	fatty, roasty	1486
19	(Z)-2-nonenal	140.22	green, fatty	1494
20	benzaldehyde [d]	106.12	almond-like	1515
21	*(E)*-2-nonenal	140.22	fatty, cardboard-like	1524
22	linalool	154.25	flowery	1539
23	1-octanol	130.23	soapy, green	1545
24	2-methylpropanoic acid	88.15	cheesy	1562
25	(*E*,*Z*)-2,6-nonadienal	138.21	cucumber-like, fatty	1574
26	acetophenone [d]	120.15	marzipan-like, flowery	1611
27	*(E)*-2-decenal	154.25	green, fatty	1616
28 a/b	2- and 3-methylbutanoic acid	102.13	rancid, cheesy	1662
29	alpha terpineol [d]	154.25	eucalyptus-like, mint-like	1675
30	(*E*,*E*)-2,4-nonadienal	138.21	fatty, deep fried	1696
31	2-undecanol [d]	172.31	soapy	1706
32	(*E*,*E*)-2,4-decadienal	152.23	fatty	1800
33	2-methoxyphenol	124.14	smoky, ham-like	1848
34	2-phenylethanol	122.16	rose-like, flowery	1897
35	4-methylhexanoic acid	130.18	sweaty, fishy	2011
36	4-hydroxy-2,5-dimethyl-3(2*H*)-furanone	128.13	caramel-like	2026
37	octanoic acid	144.21	green, soapy	2043
38	4-methylphenol	108.14	fecal	2073
39	3-hydroxy-4,5-dimethylfuran-2(5*H*)-one	128.13	maggi-like, celery-like	2194
40	undecanoic acid	186.29	soapy, coriander-like	2323

[a] Consecutive numbering of odorants according to their retention indices on capillary column DB-FFAP. [b] Odorant was identified by comparison of its odor quality and intensity, retention indices on capillary DB-FFAP as well as mass spectra (EI mode) with data of reference compounds. [c] Odor quality perceived at the odor detection port. [d] Odorant not determined in the cocoa pulp extracts with a factor of FD>2
